# Supplementary material for: Alcohol Use Disorder and Smoking-Associated Molecular Alterations in Human Prefrontal Cortex in Single Nucleus (sn) RNA-Seq
Source: bioRxiv. 2026 Sep 29:2026.09.24.753990. Preprint. [Version 1] doi: 10.64898/2026.09.24.753990 (PMC13648433; doi:10.64898/2026.09.24.753990)
Supplement: Supplementary file 1 [file media-1.pdf]

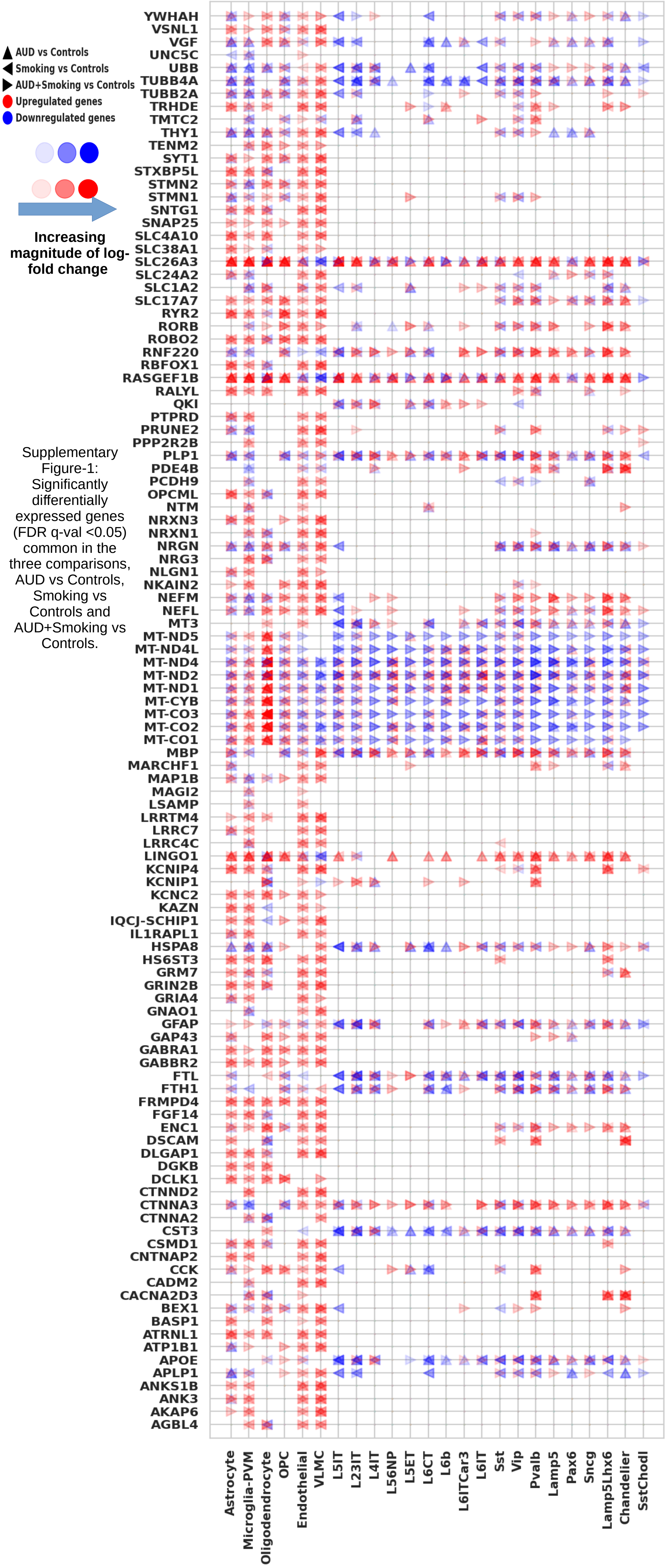

Supplementary Figure-1: Significantly differentially expressed genes (FDR q-val <0.05) common in the three comparisons, AUD vs Controls, Smoking vs Controls and AUD+Smoking vs Controls.
